# Evaluating Factor VIII Concentrates Using Clot Waveform Analysis

**DOI:** 10.3390/jcm13133857

**Published:** 2024-06-30

**Authors:** Hideo Wada, Katsuya Shiraki, Takeshi Matsumoto, Hideto Shimpo, Yumi Sakano, Hiroko Nishii, Shigehisa Tamaki, Kei Suzuki, Isao Tawara, Yoshiki Yamashita, Motomu Shimaoka

**Affiliations:** 1 Associated Department with Mie Graduate School of Medicine, Mie Prefectural General Medical Center, Yokkaichi 510-0885, Japan; katsuya-shiraki@mie-gmc.jp; 2 Department of General Medicine, Mie Prefectural General Medical Center, Yokkaichi 510-0885, Japan; 3 Department of Transfusion Medicine and Cell Therapy, Mie University Hospital, Tsu 514-8507, Japan; matsutak@clin.medic.mie-u.ac.jp; 4 Mie Prefectural General Medical Center, Yokkaichi 510-0885, Japan; hideto-shimpo@mie-gmc.jp; 5 Department of Molecular Pathobiology and Cell Adhesion Biology, Mie University Graduate School of Medicine, Tsu 514-8507, Japan; ysakano@clin.medic.mie-u.ac.jp (Y.S.); nishiihiroko@clin.medic.mie-u.ac.jp (H.N.); motomushimaoka@gmail.com (M.S.); 6 Department of Hematology, Japanese Red Cross Ise Hospital, Ise 516-8512, Japan; stamaki@ise.jrc.or.jp; 7 The Advanced Emergency and Critical Care Center, Mie University Hospital, Tsu 514-8507, Japan; keis@med.mie-u.ac.jp; 8 Department of Hematology and Oncology, Mie University Graduate School of Medicine, Tsu 514-8507, Japan; itawara@clin.medic.mie-u.ac.jp (I.T.); yamayamafan4989@yahoo.co.jp (Y.Y.)

**Keywords:** CWA, APTT reagent, sTF/FIX assay, FVIII, EHL-FVIII

## Abstract

**Background/Objectives**: FVIII reagent activity varies across different assays, as well as activated partial thromboplastin time (APTT) reagents. The hemostatic ability of various FVIII reagents was examined via clot waveform analysis (CWA). **Methods**: APTT was measured using 12 APTT reagents, a small amount of tissue factor-induced FIX activation (sTF/FIXa) and a small amount of thrombin time (sTT) in order to examine 10 FVIII reagents and reference plasma (RP) using CWA. FVIII activity was measured using CWA-APTT, a chromogenic assay, or CWA-sTT. **Results**: Although the peak time (PT) and peak height (PH) of the CWA-APTT were markedly different in different FVIII reagents using several APTT reagents, the PTs of CWA-APTT were generally normal or shortened and the PHs of CWA-APTT were generally lower than those of RP. The FVIII activity varied, as evaluated using APTT, and was higher when using the CWA-sTT method than the APTT or chromogenic methods. CWA-sTT showed an elevated second peak of first DPH in all FVIII reagents, and both CWA-sTF/FIXa and CWA-sTT were enhanced using APTT reagents. **Conclusions:** Our evaluation of the hemostatic ability of FVIII reagents varied among APTT reagents. CWA-sTT can be used to further evaluate the hemostatic ability of an FVIII concentrate based on thrombin burst.

## 1. Introduction

Hemophilia A is a bleeding disorder in which hemostatic ability is decreased due to coagulation factor VIII (FVIII) deficiency [1,2]. Advances in the treatment and prophylaxis of bleeding and joint damage have reduced its associated mortality [3,4,5]. Prophylaxis with FVIII concentrate significantly reduces the risk of hemophilic arthropathy and is the gold standard for managing severe hemophilia A [6]. Recent progress in hemophilia management [7] includes extended half-life FVIII (EHL-FVIII) [8], FVIII mimetics (e.g., emicizumab) [9,10], non-factor therapies (e.g., tissue factor pathway inhibitor) [11,12], and gene therapy [13,14]. EHL-FVIII reduces the necessary number of injections and substantially improves treatment options for patients with hemophilia A [8,15]. Recently, efanesoctocog alfa (Altuviiio^®^, BIVV001, Swedish Orphan Biovitrum AB ([SOBI]-Sanofi), which is a new class of rFVIII molecule with an EHL, has become available for prophylaxis against hemophilia [16,17]. Evaluating the hemostatic ability of FVIII concentrate including EHL- and standard half-life-(SHL)-FVIII requires clotting time assays.

Although the activated partial thromboplastin time (APTT) is useful in screening for clotting factor deficiencies (e.g., hemophilia A [18]) and the presence of inhibitors [19,20], as well as in monitoring anticoagulant therapy (e.g., heparin) [21], it is not suitable for patients treated with emicizumab [22,23]. The FVIII activity in hemophilic patients treated with EHL-FVIII, including polyethylene glycol (PEG)-FVIII concentrate, varied across various APTT reagents [24,25], suggesting that some may not be useful for monitoring hemophilic patients treated with some FVIII concentrates. Hypercoagulability has recently been reported in hemophilic patients treated with FVIII concentrates [26]. Many optical automatic coagulation analyzers can demonstrate the clot formation curve in APTT [27,28,29], a small amount of tissue factor-induced FIX activation assay (sTF/FIXa) [30] and a small amount of thrombin time (sTT) [31]. This analysis of the coagulation curve is called a clotting waveform analysis (CWA) [32]. Furthermore, a new FVIII assay using sTT is reportedly useful for measuring FVIII levels in patients treated with emicizumab [33]. In addition, CWA-sTF/FIXa and CWA-sTT may reflect thrombin burst, which mainly depends on the activation of FV, FVIII, and FXI [34,35].

In this study, the hemostatic abilities of the ten FVIII concentrates were examined via CWA using twelve APTT reagents. Furthermore, the relationship between FVIII concentrates and thrombin burst was examined via CWA-sTF/FIXa and CWA-sTT.

## 2. Materials and Methods

### 2.1. APTT Reagents

I, HemosIL APTT-SP (APTT-SP; Werfen, Bedford, MA, USA); II, HemosIL SynthASil (APTT-SS; Werfen); III, STA Cephascreen (Diagnostica Stago S.A.S., Asnières-sur-Sreine, France); IV, Coagpia APTT-N (APTT-N; SEKISUI MEDICAL Co., Ltd., Tokyo, Japan); V, APTT ACT and Pathromtin^®^ SL (APTT PSL; Siemens Healthcare Diagnostics Products GmbH, Marburg, Germany); VI, C.K. Prest (Diagnostica Stago S.A.S.); VII, Thrombocheck APTT-SLA (APTT-SLA; Sysmex Corporation, Kobe, Japan); VIII, Thrombocheck APTT (TC APTT; Sysmex Corporation); IX, Dade^®^Actin^®^ FS Activated PTT Reagent (APTT FS; Sysmex Corporation); X, APTT FSL (Sysmex Corporation); XI, Activated Cephaloplastin Reagent (PTT ACT; Siemens Healthcare Diagnostics Products GmbH, Marburg, Germany); XII, STA PTT Automate (STA PTT A; Diagnostica Stago S.A.S.).

FVIII, coagulation factor FVIII, A, Cross eight MC (Japan Blood Production Organization, Tokyo, Japan); B, Confact F (Japan Blood Production Organization); C, Turoctocog alfa (Novo Nordisk Pharma, Bagsværd, Danmark); Ce, Turoctocog Alfa Pegol (Novo Nordisk Pharma); D, Octocog beta (Bayer Pharma, Leverkusen, Germany); De, Damoctocog Alfa Pegol (Bayer Pharma, Leverkusen, Germany); E, Rurioctocog alfa (Takeda Pharmaceuticals, Osaka, Japan); Ee, Rurioctocog alfa pegol; F, Lonoctocog alfa (CSL Behring K.K., Tokyo, Japan); Ge, Efraloctocog alfa (Sanofi K.K., Tokyo, Japan); e, enhanced half-life; APTT, activated partial thromboplastin time; sTT, small amount of thrombin time assay; RP, reference plasma.

The sample, reference plasma (RP, Werfen), and FVIII concentrate were diluted to 1.0 IU/mL with FVIII-deficient plasma (Werfen), which had been artificially depleted of factor VIII containing buffer and stabilizers. The residual factor VIII activity was less than or equal to 1%, whereas all other coagulation factors had normal levels. FVIII 1.0 IU adjustment was performed via the dilution based on the vial description.

In 1.0 IU/mL of RP and FVIII concentrate diluted with FVIII-deficient plasma, CWA-APTT was performed using the above-mentioned APTT reagents and an ACL-TOP^®^ system (Werfen). The changes in absorbance observed during the APTT measurement consisted of the fibrin formation curve (FFC), the first derivative peak (1st DP), and the second derivative peak (2nd DP). The 1st DP and 2nd DP correspond to coagulation velocity and acceleration, respectively. One limitation of the present study is that there is little evidence to support the use of the ACL-TOP instrument with various APTT reagents (III–XII). However, we performed all CWAs using various APTT reagents with only one instrument, the ACL-TOP. The reason for this was that the ACL-TOP shows a more superior performance than most in CWAs.

Regarding modified CWAs, the sTF/FIX assay was performed using 1 IU/mL of RP or FVIII concentrate diluted with FVIII-deficient plasma, and 2000-fold diluted HemosIL RecombiPlasTin 2G, (Werfen) with an ACL-TOP^®^ system [36]. The CWA-TT using 0.5 IU thrombin (Thrombin 500 units; Mochida Pharmaceutical Co., Ltd., Tokyo, Japan) was measured using an ACL-TOP^®^ system [31,33]. In addition, modified CWAs such as CWA-sTF/FIXa and CWA-TT were performed with and without APTT-SP [37].

FVIII activity was measured using the APTT one-stage clotting assay [38]. The activity was also measured using the 2nd DPT using APTT-SP and FVIII-deficient plasma (Werfen) in an ACL-TOP system. Additionally, the chromogenic substrate method was used with a Revohem^TM^ FVIII chromogenics system (HYPHEN BioMed, Neuville-sur-Oise, France) [39] with a CS-5100 device (Sysmex Corporation). The CWA-TT method was used with FIII-deficient plasma and an ACL-TOP system [26].

### 2.2. Statistical Analyses

Five measurements were performed in all assays. Data are expressed as the mean ± standard deviation. The significance of differences between groups was examined using Student’s *t* test. *p* values of <0.01 were considered to indicate statistical significance. All statistical analyses were performed using the Stat-Flex software program (version 6; Artec Co., Ltd., Osaka, Japan).

## 3. Results

**Figure 1:**
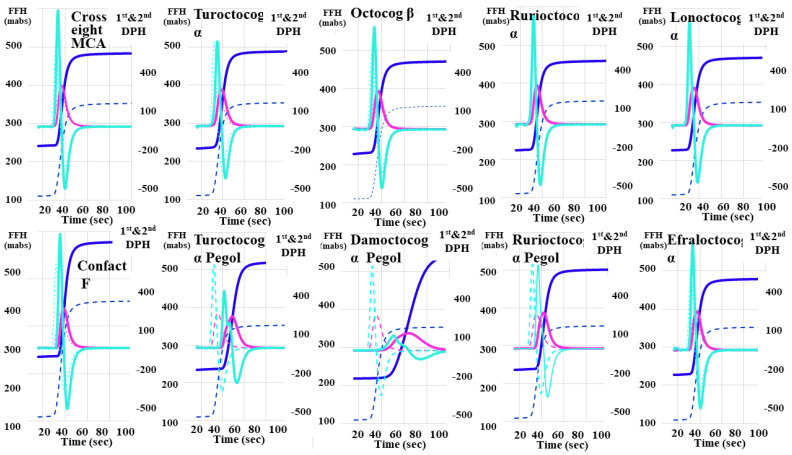
Clot waveform analysis—activated partial thromboplastin time for FVIII reagents. FVIII, coagulation factor FVIII; navy line, fibrin formation curve; FFH, fibrin formation height; pink line, 1st derivative curve (velocity); 1st DPH, first derivative peak height; light blue, 2nd derivative curve (acceleration); 2nd DPH, second derivative peak height; solid line, FVIII reagent; dotted line, reference plasma.

Regarding the CWA-APTT for the ten FVIII reagents using APTT-SP (Figure 1 and Table 1), the second DPT, first DPT, and FFT showed significant (*p* < 0.01) increases in Turoctocog α Pegol and Damoctocog α Pegol; in Turoctocog α Pegol, Damoctocog α Pegol, and Rurioctocog αpegol; and in Turoctocog α Pegol and Damoctocog α Pegol, respectively.

Meanwhile, they showed significant decreases in Cross eight MC, Octocog β, Rurioctocog α, Lonoctocog α, and Efraloctocog α; in Cross eight MC, Confact F, Octocog β, Rurioctocog α, Lonoctocog α, and Efraloctocog α; and in Cross eight MC, Octocog β, Rurioctocog α, Lonoctocog α, and Efraloctocog α, respectively, in comparison to the reference plasma. The second DPH, first DPH, and FFH showed significant (*p* < 0.01) increases in Cross eight MC, Confact F, Turoctocog α, Rurioctocog α, Lonoctocog α, and Ge, in no reagent, and in Cross eight MC, Confact F, Turoctocog α, Turoctocog α Pegol, Octocog β, Damoctocog α Pegol, Rurioctocog α, Lonoctocog α, and Efraloctocog α. Meanwhile, they showed significant decreases in Turoctocog α Pegol, Octocog β, Damoctocog α Pegol, Lonoctocog α, and Efraloctocog α; in Turoctocog α Pegol and Damoctocog α Pegol; and in no reagent, respectively.

Regarding the first DPT of the CWA-APTT for the ten FVIII concentrates using various APTT reagents (Table 2), the first DPT of all FVIII concentrates using APTT-SS, APTT-N, APTT PSL, C.K. Prest, APTT-SLA, and APTT FSL was normal or shortened in comparison to RP; however, a prolongation of >10 s in the first DPT was observed in Turoctocog α Pegol and Damoctocog α Pegol using APTT-SS or STA PTT A. For most FVIII concentrates using most APTT reagents, except for APTT-SS or APTT-N (Table 2), the first DPH of CWA-APTT was lower than that of RP. In particular the CWA-APTT for Damoctocog α Pegol using APTT-SS or STA PTT A saw a <100 mm reduction in absorbance in comparison to RP, while significant decreases were observed in Cross eight MC, Octocog β, Rurioctocog alfa, Lonoctocog α, and Efraloctocog α. Using a chromogenic assay, the FVIII activity was similar from 1.0 IU/mL to 1.04 IU/mL for all reagents (Table 3), but that measured via APTT using APTT-SP (APTT reagent I) varied, being significantly lower in Turoctocog α Pegol and Damoctocog α Pegol. The FVIII activity induced by CWA-sTT was high, ranging from 1.25 IU/mL to 1.78 IU/mL.

**Figure 2:**
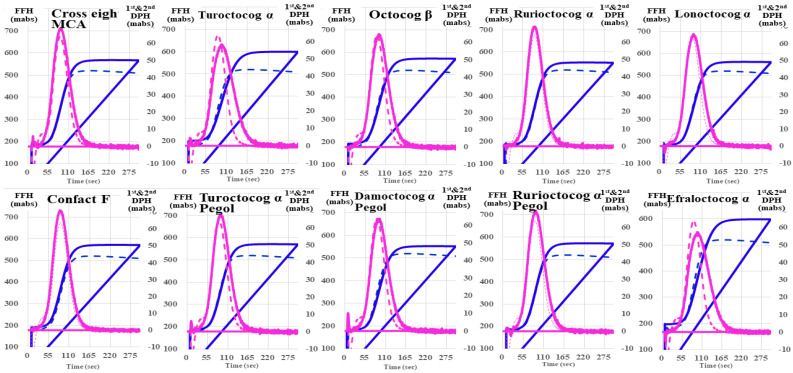
Clot waveform analysis—small amount of tissue factor-induced FIX activation assay for FVIII reagents in comparison with reference plasma. FVIII, coagulation factor FVIII; navy line, fibrin formation curve; FFH, fibrin formation height; pink line, 1st derivative curve (velocity); 1st DPH, first derivative peak height; solid line, FVIII reagent; dotted line, reference plasma.

**Figure 3:**
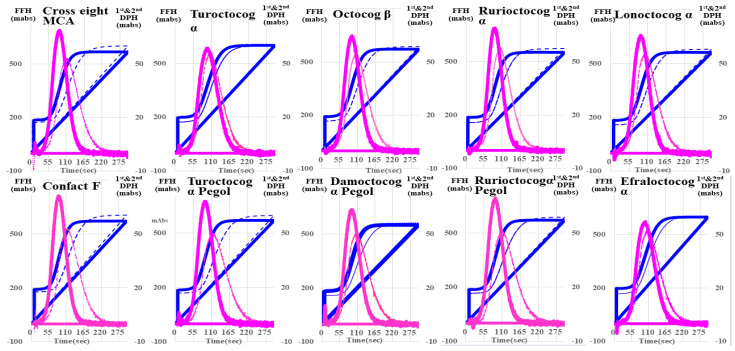
Clot waveform analysis—small amount of tissue factor-induced FIX activation assay for FVIII reagents with and without APTT reagent. FVIII, coagulation factor FVIII; APTT, activated partial thromboplastin time; navy line, fibrin formation curve; FFH, fibrin formation height; pink line, 1st derivative curve (velocity); 1st DPH, first derivative peak height; light blue, 2nd derivative curve (acceleration); 2nd DPH, second derivative peak height; solid line, with APTT reagent; dotted line, without APTT reagent.

Regarding the CWA-STF/FIXa of the ten FVIII concentrates, there were no significant differences in them or between each concentrate and the RP (Figure 2). The FFH of CWA-sTF/FIXa was slightly higher for each FVIII concentrate than for RP. The first DPT and FFT were significantly shorter for each FVIII concentrate with APTT reagent in comparison to those without (Figure 3).

**Figure 4:**
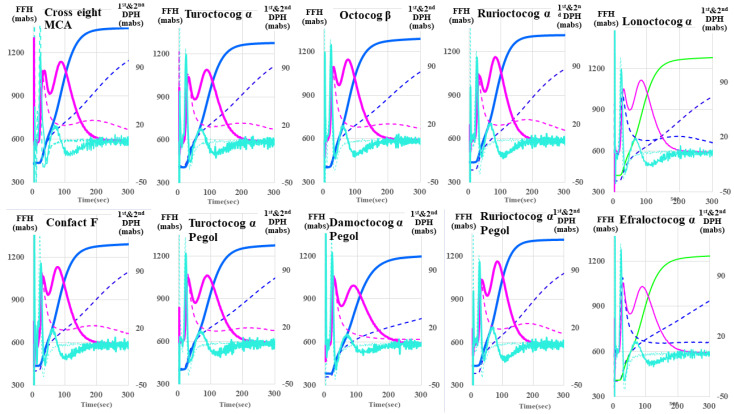
Clot waveform analysis—small amount of thrombin time for FVIII reagents. FVIII, coagulation factor FVIII; navy line, fibrin formation curve; FFH, fibrin formation height; pink line, 1st derivative curve (velocity); 1st DPH, first derivative peak height; light blue, 2nd derivative curve (acceleration); 2nd DPH, second derivative peak height; solid line, FVIII reagent; dotted line, reference plasma.

**Figure 5:**
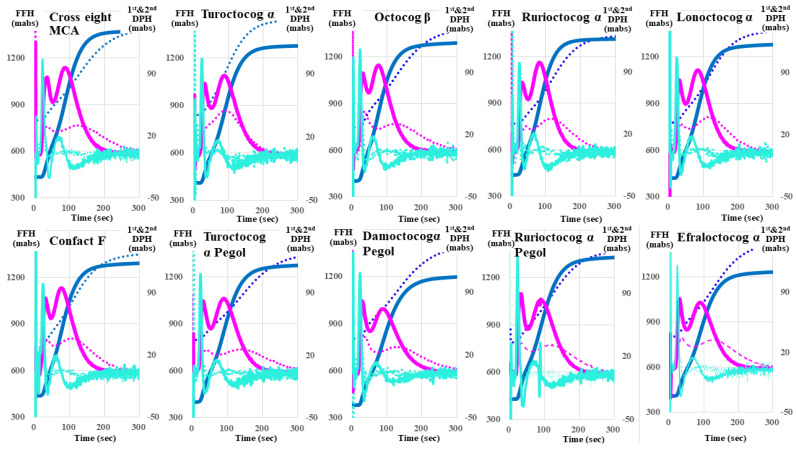
Clot waveform analysis—small amount of thrombin time for FVIII reagents with and without APTT reagent. FVIII, coagulation factor FVIII; APTT, activated partial thromboplastin time; navy line, fibrin formation curve; FFH, fibrin formation height; pink line, 1st derivative curve (velocity); 1st DPH, first derivative peak height; light blue, 2nd derivative curve (acceleration); 2nd DPH, second derivative peak height; solid line, with APTT reagent; dotted line, without APTT reagent.

Regarding the CWA-sTT of the ten FVIII concentrates, although there were no significant differences, the second peak height of the first DP and FFH on the sTT was markedly higher in each FVIII reagent than in the RP (Figure 4). The second peak height of the 1st DP on the sTT was markedly higher in each FVIII concentrate with APTT reagent than in those without (Figure 5).

## 4. Discussion

The hemostatic ability of hemophilic patients treated with FVIII concentrates is usually monitored via APTT or FVIII activity [40]. Several methods are used to determine FVIII, including activity assays such as the one-stage method using APTT, the chromogenic substrate assay [41,42], CWA-sTF/FIXa [36], and CWA-TT [26]. FVIII concentrates are usually classified as SHL-FVIII or EHL-FVIII. Although EHL-FVIII can reduce the administration time of FVIII concentrates [43,44], several of them showed a marked variability in the APTT assays; this can be intensified in an APTT reagent-specific manner, and has attracted the attention of many researchers [24,45,46,47]. The APTT assay has recently been used to further evaluate hemostatic ability. CWA-APTT consists of a peak time, using a routine APTT and a peak height which, thus far, have rarely been reported [10].

The present CWA-APTT study using APTT-SP (I) also showed a marked prolongation of the first DPT in Turoctocog Alfa and Damoctocog Alfa Pegol. Both FVIII concentrates are EHL-FVIII concentrates, which bind to PEG (PEG-FVIII). It has previously been reported that the FVIII activity in patients treated with EHL-FVIII, including PEG-FVIII, varied among different APTT reagents [24]. APTT-SP consists of a synthetic phospholipid and colloidal silica, and the PEG moiety on EHL-FVIII appears to interact with silica-based APTT reagents, leading to a prolonged APTT [24,48]. A marked reduction in first DPH was only observed in Damoctocog Alfa Pegol (De), and a marked abnormality of CWA-APTT was not observed in Rurioctocog alfa pegol (Ee). These findings suggest that measurements of both peak time and height are useful for evaluating hemostatic abnormalities and that APTT prolongation may not solely be due to PEG-FVIII-silica interactions. The CWA-APTT for various APTT reagents showed that the peak times and heights varied among the 12 APTT-reagents. The first DPT of CWA-PTT, using APTT-SS, APTT-N, C.K. Prest, APTT-SLA, or APTT FSL, did not show markedly prolonged values and neither did the first DPH of CWA-APTT using APTT reagents APTT-SS or APTT-N, suggesting that the latter APTT reagent may be useful for monitoring patients treated with FVIII concentrates.

CWA-sTF/FIXa and CWA-TT showed no marked differences among the FVIII concentrates, suggesting that these assay systems may be more useful for their monitoring. In addition, APTT assay systems, such as incubation with the APTT reagent, may be responsible for the differences among various FVIII concentrates. Furthermore, the addition of APTT reagents shortened PT and increased PH in CWA-sTF/FIXa and markedly elevated the second peak of first DP of CWA-sTT. These effects suggest that thrombin burst [49,50] may affect the hemostatic ability of FVIII concentrates in hemophilic patients. Under laboratory examination, APTT reagents play an important role in thrombin burst; platelets may also perform an important role in this regard in physiological and pathological states [51,52]. Thrombin burst has generally been evaluated via thromboelastography (TEG) [53] and the thrombin generation test (TGT) [34,35]. It was recently reported that CWA-sTT can reflect thrombin burst [31,51].

The chromogenic assay showed a similar FVIII activity among the ten FVIII concentrates. FVIII activity in the APTT one-stage assay varied and was significantly low, especially for Turoctocog Alfa Pegol and Damoctocog Alfa Pegol, while that in CWA-sTT varied and was higher than that in the other two assays. As FVIII activity via CWA-APTT or CWA-sTT varies depending on the APTT reagent, physicians need to know the APTT reagent used in their laboratory. However, chromogenic assays cannot detect the effects of thrombin burst. The FVIII activity in hemophilic patients treated using recent EHL-FVIII prophylaxis therapies tended to be high [8,54]; in particular, efanesoctocog alfa, a von Willebrand factor (VWF)-independent recombinant FVIII concentrate, may elevate FVIII activity by more than 100–150% in hemophilic patients [16,55]. Elevated FVIII levels were reported to be associated with a high risk of venous thromboembolism [56,57]. Therefore, hemophilic patients treated with FVIII concentrate are also considered at risk for thrombosis [58,59]. CWA-sTF/FIXa and CWA-sTT reportedly show hypercoagulability in hemophilic patients treated with FVIII concentrate.

## 5. Conclusions

The hemostatic ability of FVIII concentrates varied among APTT reagents; FVIII assays also varied with and without the APTT reagent. CWA-sTF/FIX and CWA-sTT can be used to evaluate the hemostatic ability of FVIII concentrates based on thrombin burst.

## Figures and Tables

**Table 1:** Clot waveform analysis of APTT for FVIII reagents.

	Second Derivative		First Derivative		Fibrin Formation Curve	
	PT (s)	PH (mabs)	PT (s)	PH (mabs)	PT (s)	PH (mabs)
Cross eight MC	34.9 ± 1.0 ** (↓)	733 ± 97.7 ** (↑)	38.2 ± 1.14 ** (↓)	266 ± 10.1 ^NS^	39.2 ± 1.2 ** (↓)	427 ± 9.8 *** (↑)
Confact F	35.9 ± 0.6 ^NS^	798 ± 46.5 *** (↑)	39.1 ± 0.8 ** (↓)	271 ± 9.2 ^NS^	40.1 ± 0.8 *	574 ± 8.5 *** (↑)
Turoctocog α	36.1 ± 0.6 ^NS^	621 ± 31.6 * (↑)	40.3 ± 0.7 ^NS^	251 ± 2.5 ^NS^	41.0 ± 0.6 ^NS^	420 ± 6.6 *** (↑)
Turoctocog α Pegol	43.2 ± 0.9 *** (↑)	368 ± 15.5 *** (↓)	50.9 ± 1.1 *** (↑)	204 ± 7.1 *** (↓)	51.2 ± 1.0 *** (↑)	352 ± 47.8 ^NS^
Octocog β	35.0 ± 0.7 ** (↓)	684 ± 34.9 ** (↑)	38.4 ± 0.7 *** (↓)	258 ± 7.2 ^NS^	39.4 ± 0.7 ** (↓)	538 ± 17.0 *** (↑)
Damoctocog α Pegol	55.5 ± 7.1 *** (↑)	93.7 ± 10.1 *** (↓)	69.3 ± 8.1 *** (↑)	115 ± 5.5 *** (↓)	70.2 ± 8.1 *** (↑)	530 ± 3.4 *** (↑)
Rurioctocog α	33.6 ± 0.6 *** (↓)	719 ± 23.1 *** (↑)	36.9 ± 0.7 *** (↓)	263 ± 3.8 ^NS^	37.9 ± 0.7 *** (↓)	406 ± 6.5 *** (↑)
Rurioctocog α pegol	38.3 ± 0.8 ^NS^	557 ± 16.7	43.5 ± 0.9 ** (↑)	237 ± 5.2 *	44.1 ± 0.8 ^NS^	503 ± 10.3 *** (↑)
Lonoctocog α	32.1 ± 0.3 *** (↓)	697 ± 8.7 ** (↑)	36.3 ± 0.4 *** (↓)	254 ± 2.0 ^NS^	37.0 ± 0.4 *** (↓)	412 ± 7.5 *** (↑)
Efraloctocog α	34.5 ± 0.8 ** (↓)	692 ± 26.0 ** (↑)	38.7 ± 1.0 ** (↓)	254 ± 6.5 ^NS^	39.5 ± 0.9 ** (↓)	432 ± 4.8 *** (↑)
Reference plasma	37.3 ± 1.1	536 ± 66.8	41.2 ± 0.9	260 ± 20.1	42.1 ± 1.1	342 ± 9.8

FVIII—coagulation factor FVIII; APTT—activated partial thromboplastin time; PT—peak time; PH—peak height; *, **, ***, and ^NS^—*p* < 0.05, *p* < 0.01, *p* < 0.001, and not significant, respectively, in comparison with reference plasma; (↑)—significantly (*p* < 0.01) increased peak time or height; (↓)—significantly (*p* < 0.01) decreased peak time or height; underline—prolongation of >10 s or reduction of >100 mm absorbance in comparison with reference plasma.

**Table 2:** First derivative peak times (a) or peak heights (b) of clot waveform analysis—activated partial thromboplastin time in various FVIII reagents.

APTT Reagents	FVIII Concentrates										
	Cross Eight MC	Confact F	Turoctocog α	Turoctocog α Pegol	Octocog β	Damoctocog α Pegol	Rurioctocog α	Rurioctocog α Pegol	Lonoctocog α	Efraloctocog α	RP
	(a) Peak Time (s)										
APTT-SP	38.1 ± 1.1	39.1 ± 0.8	40.3 ± 0.7	50.9 ± 1.1 *	38.4 ± 0.7 *	69.3 ± 8.1 *	36.9 ± 0.7 *	43.5 ± 0.9	36.3 ± 0.4 *	38.7 ± 1.0	41.2 ± 0.9
APTT-SS	30.2 ± 0.6 *	30.0 ± 0.4 *	27.0 ± 0.3 *	30.6 ± 0.4 *	30.5 ± 0.7	32.0 ± 0.2	26.4 ± 0.5 *	30.0 ± 0.3 *	30.0 ± 0.5 *	30.9 ± 0.8 *	39.6 ± 0.9
STA Cepha-screen	36.4 ± 0.6	39.7 ± 0.4 *	39.0 ± 0.4 *	38.3 ± 0.3 *	34.9 ± 0.2 *	32.6 ± 0.3 *	35.2 ± 0.4	37.8 ± 0.4 *	35.5 ± 0.4	36.9 ± 0.7	35.5 ± 0.8
APTT-N	27.9 ± 0.3 *	29.6 ± 0.4 *	27.4 ± 0.1 *	26.3 ± 0.4 *	27.7 ± 0.3 *	27.2 ± 0.5 *	25.0 ± 0.3 *	27.8 ± 0.4 *	29.2 ± 0.2 *	28.0 ± 0.3 *	33.9 ± 0.5
APTT PSL	44.0 ± 0.4 *	42.9 ± 0.6 *	44.0 ± 0.3 *	44.3 ± 0.3 *	40.7 ± 0.2 *	43.5 ± 3.4	42.3 ± 0.5 *	41.9 ± 0.2 *	42.4 ± 0.9 *	49.5 ± 0.6 *	46.4 ± 0.6
C.K. Prest	33.9 ± 0.3 *	32.2 ± 0.7 *	34.3 ± 0.4 *	43.7 ± 0.3 *	31.4 ± 0.4 *	36.4 ± 0.3	32.5 ± 0.6 *	34.6 ± 0.4 *	32.3 ± 0.3 *	33.1 ± 0.5 *	36.9 ± 0.6
APTT-SLA	30.1 ± 0.5 *	29.8 ± 0.4 *	30.4 ± 0.3 *	30.9 ± 0.3 *	27.7 ± 0.3 *	25.7 ± 0.3 *	28.2 ± 0.5 *	31.4 ± 0.5	28.8 ± 0.3 *	30.6 ± 0.4 *	31.9 ± 0.2
TC APTT	35.9 ± 0.4	35.0 ± 0.4 *	38.1 ± 0.3 *	36.8 ± 0.2 *	31.3 ± 0.4 *	28.6 ± 0.4 *	34.3 ± 0.6	37.5 ± 0.4 *	34.9 ± 0.2 *	36.9 ± 0.3 *	35.8 ± 0.3
APTT FS	36.2 ± 0.5	36.0 ± 0.6	38.2 ± 0.3 *	39.8 ± 0.4 *	33.1 ± 0.3 *	29.8 ± 0.3 *	36.3 ± 0.4	40.2 ± 0.4 *	38.9 ± 0.1 *	38.2 ± 0.5 *	36.8 ± 0.1
APTT FSL	30.0 ± 0.7 *	29.9 ± 0.7 *	26.3 ± 0.2 *	28.1 ± 0.3 *	27.4 ± 0.5 *	26.8 ± 0.3 *	24.9 ± 0.2 *	27.9 ± 0.5 *	27.3 ± 0.4 *	28.6 ± 0.3 *	36.3 ± 0.6
PTT ACT	37.2 ± 0.5 *	36.2 ± 0.5 *	40.9 ± 1.3 *	42.2 ± 0.6 *	34.9 ± 0.2	29.7 ± 0.4 *	38.2 ± 0.5 *	40.7 ± 0.5 *	39.4 ± 0.3 *	40.5 ± 0.3 *	33.1 ± 0.8
STA PTT A	39.8 ± 0.5	37.9 ± 0.4	42.0 ± 0.6	56.7 ± 0.4 *	33.9 ± 0.3 *	74.8 ± 0.5 *	37.5 ± 0.4	44.1 ± 0.3	41.3 ± 0.4	39.9 ± 0.3	41.7 ± 3.3
	**(b) Peak heights (mabs)**										
APTT-SP	266 ± 9.0	270 ± 8.2	251 ± 2.4	204 ± 6.2 *	259 ± 6.3	115 ± 4.9 *	263 ± 3.4	237 ± 4.5	255 ± 1.9	255 ± 5.7	260 ± 17.9
APTT-SS	201 ± 5.0	202 ± 5.6	192 ± 6.4	188 ± 2.9	188 ± 1.5	193 ± 1.6	199 ± 3.0	187 ± 3.7	200 ± 2.7	204 ± 2.6	188 ± 22.6
STA Cepha-screen	251 ± 7.1	241 ± 6.1 *	227 ± 5.7 *	230 ± 2.4 *	241 ± 1.5 *	258 ± 1.6	236 ± 5.5 *	231 ± 4.8 *	231 ± 1.1 *	265 ± 4.4	271 ± 12.2
APTT-N	220 ± 5.0	217 ± 5.4	221 ± 4.0	212 ± 4.1	220 ± 3.1	221 ± 4.9	229 ± 4.3	217 ± 3.2	208 ± 1.1	227 ± 6.3	221 ± 10.9
APTT PSL	189 ± 4.0 *	195 ± 5.0 *	180 ± 6.8 *	144 ± 10.7 *	196 ± 4.2 *	155 ± 3.2 *	184 ± 3.7 *	169 ± 4.4 *	180 ± 1.9 *	192 ± 2.1 *	212 ± 5.2
C.K. Prest	153 ± 3.5	158 ± 3.5	142 ± 3.1 *	128 ± 2.5 *	168 ± 4.8	153 ± 4.0	150 ± 3.7	138 ± 3.3	155 ± 2.5	156 ± 4.8	169 ± 13.5
APTT-SLA	283 ± 3.1 *	285 ± 5.4 *	274 ± 4.3 *	271 ± 6.3 *	295 ± 4.1 *	320 ± 7.7	280 ± 2.7 *	276 ± 5.0 *	272 ± 3.6 *	306 ± 3.1 *	332 ± 4.8
TC APTT	251 ± 3.2 *	260 ± 6.3	247 ± 3.3 *	246 ± 2.4 *	257 ± 4.8	283 ± 3.1	248 ± 3.6 *	261 ± 4.2	240 ± 1.3 *	264 ± 3.4	272 ± 4.7
APTT FS	255 ± 4.7 *	252 ± 5.0 *	240 ± 6.3	251 ± 1.6 *	291 ± 6.0	307 ± 5.4	249 ± 4.4 *	250 ± 3.5 *	253 ± 1.6 *	276 ± 3.4 *	302 ± 3.9
APTT FSL	253 ± 4.6 *	256 ± 5.9 *	256 ± 4.9	256 ± 2.4 *	268 ± 5.8	297 ± 3.1 *	261 ± 3.1 *	260 ± 3.5 *	252 ± 1.9 *	279 ± 3.9	280 ± 6.0
PTT ACT	299 ± 6.3 *	298 ± 5.9 *	271 ± 3.8 *	295 ± 2.7 *	312 ± 4.3	352 ± 4.2	294 ± 6.6 *	279 ± 4.3 *	281 ± 1.9 *	319 ± 2.4	338 ± 11.3
STA PTT A	218 ± 6.2	228 ± 7.8	202 ± 4.7 *	153 ± 1.9 *	279 ± 5.6	131 ± 4.0 *	207 ± 4.1 *	180 ± 3.7 *	219 ± 1.6 *	242 ± 33.3	249 ± 12.5

[APTT reagents] APTT-SP, HemosIL APTT-SP; APTT-SS, HemosIL SynthASil; APTT-N, Coagpia APTT-N; APTT PSL, PTT ACT and Pathromtin ^®^ SL; APTT-SLA, Thrombocheck APTT-SLA; TC APTT, Thrombocheck APTT; APTT FS, Dade^®^ Actin^®^ FSL Activated Cephaloplastin Reagent; APTT FSL, Dade^®^ Actin ^®^ FSL Activated PTT Reagent; PTT ACT, Activated Cephaloplastin Reagent; STA PTT A, STA PTT Automate; FVIII, coagulation factor FVIII, APTT, activated partial thromboplastin time reagents; RP, reference plasma; underline, significantly decreased peak time (a) or height (b); double underline, prolongation of 10 s in comparison with RP (a) or <100 mm absorbance in comparison with RP (b); *, *p* < 0.001 in comparison to RP.

**Table 3:** FVIII activity in in 1.0 U/mL of each FVIII reagent.

Method	Cross Eight MC	Confact F	Turoctocog α	Turoctocog α Pegol	Octocog β	Damoctocog α Pegol	Rurioctocog α	Rurioctocog α Pegol	Lonoctocog α	Efraloctocog α
Chromogenic assay (IU/mL)	1.00	1.01	1.01	1.00	1.04	1.03	1.00	1.03	1.04	1.00
APTT (IU/mL)	1.04	1.05	0.80	0.48	0.96	0.12	1.01	0.81	1.03	1.02
sTT (IU/mL)	1.60	1.78	1.34	1.40	1.25	1.20	1.51	1.31	1.44	1.33

FVIII, coagulation factor FVIII; APTT, activated partial thromboplastin time; sTT, small amount of thrombin time assay.

## Data Availability

The data presented in this study are available on request to the corresponding author. The data are not publicly available due to privacy restrictions.
